# Stress tolerance of *Xerocomus badius* and its promotion effect on seed germination and seedling growth of annual ryegrass under salt and drought stresses

**DOI:** 10.1186/s13568-020-01172-7

**Published:** 2021-01-07

**Authors:** Binghua Liu, Xinghong Liu, Fangchun Liu, Hailin Ma, Bingyao Ma, Lin Peng

**Affiliations:** 1 Shandong Academy of Forestry, 42, East Wenhua Road, Shandong 250014 Jinan, China; 2 Economic Forest Products Quality Inspection Test Center of State Forestry Administration (Jinan), Shandong 250014 Jinan, China; 3 Shandong Engineering Research Center for Ecological Restoration of Forest Vegetation, Shandong 250014 Jinan, China

**Keywords:** Drought stress, Mycelium growth, Salt stress, Seed germination, Seedling growth, *Xerocomus badius*

## Abstract

Comparative evaluations were conducted to assess the effects of different pH levels, NaCl-induced salt stress, and PEG-induced drought stress on the mycelial growth of *Xerocomus badius*. The results showed that *X. badius* mycelium grew well at a wide pH range of 5.00 ~ 9.00. Although the mycelium remained viable, mycelial growth of *X. badius* was significantly inhibited with increasing salt and drought stresses. Furthermore, a soilless experiment in Petri dishes was performed to investigate the potential of *X. badius* to induce beneficial effects on seed germination and seedling growth of annual ryegrass (*Lolium multiflorum* Lam.) under salt and drought stresses. Seed priming with *X. badius* enhanced the seedling growth of *L. multiflorum* Lam. under NaCl-induced salt stress and PEG-induced drought stress. However, *X. badius* did not significantly improve the seed germination under non-stress and mild stress conditions. It suggested that *X. badius* inoculation with seeds was not essential for seed germination under non-stress and mild stress conditions, but contributed highly to seedling growth under severe stress conditions. Therefore, seed priming with *X. badius* on ryegrass could be an effective approach to enhance plant tolerance against drought and salt stresses. *X. badius* could be a good candidate for the inoculation of ectomycorrhizal plants cultivation programs in mild saline and semiarid areas.

## Introduction

Abiotic and biotic stresses influence plant growth, survival and productivity. Drought and high salinity are the two most important environmental factors that negatively affect seed germination, seedling growth and development, and ultimately influence crop yield, food quality and global food security. Application of stress tolerant plant growth promoting fungi (PGPF) may enhance crop seed germination, seedling establishment, plant growth, and productivity under adverse environmental conditions (de Zelicourt et al. 2013; Guerrero-Galán et al. 2019; Hossain et al. 2017; Kumar and Verma 2018; Tomer et al. 2016; Vijayabharathi et al. 2016; Vimal et al. 2017; Yan et al. 2019).

Mycorrhizal fungi are one of the commonly occurring microorganisms in soil, and more than 80% of land plants naturally establish mutualistic symbiotic relationships with these fungi (Bonfante and Genre 2010). Mycorrhizal fungi play an increasing vitally important role in host plants growth promotion, in inducing plant stress tolerance and agricultural sustainability under various environmental stress conditions (Behie and Bidochka 2014; Bonfante and Genre 2010; Courty et al. 2010; Garcia et al. 2016; Hossain et al. 2017; Javeria et al. 2017; Shen et al. 2018; Yan et al. 2019). Ectomycorrhizal (ECM) fungi, about 7000 to 10,000 species in the world, play a vital role in plants nutrient cycle by establishing mutual symbiosis with plants’ roots (Becquer et al. 2019; Cairney 2012; Taylor and Alexander 2005). Application of the beneficial mycorrhizal fungi in agricultural practices promises to be a fundamental tool for sustainability of crop production (Owen et al. 2015; Prasad et al. 2016; Tomer et al. 2016). In order to develop controlled ectomycorrhization practices that are suitable for the inoculation of field plants and are efficient in promoting host plants’ growth under specific environmental conditions, it is necessary to isolate potential ECM fungi and evaluate their biological, physiological and symbiotic characteristics, as well as the specificity that they have with certain hosts, under the controlled laboratory conditions.

Here, we investigated the effects of different pH levels, salt stress and drought stress on mycelial growth of ECM fungus *Xerocomus badius* (synonyms for *Boletus badius* and *Imleria badi*a) (Species Fungorum 2019) in the tolerance test. Based on the findings from the tolerance test with *X. badius* and the verified mutualistic symbiosis between *Lolium multiflorum* Lam. and *X. badius* driven by seed inoculation (Liu et al. 2019), we propose that *X. badius* is expected to enhance stress tolerance of *L. multiflorum* Lam. under drought and salt stresses. Therefore, symbiotic tests were carried out to investigate the effect of seed-priming with the spore suspensions of *X. badius* on seed germination and seedling growth of *L. multiflorum* Lam. under different NaCl-induced salt stress and PEG-induced drought stress conditions.

The general, objective of this study was (1) to evaluate the stress tolerance of *X. badius* under different pH values, salt concentrations and drought, that could be helpful in determining optimized protocols for the vegetative propagation under laboratory conditions, and to (2) verify the improvement effect of seed priming with fungus suspension on seed germination and seedling growth of *L. multiflorum* Lam. under drought and salt stressed conditions, that could have important implications for the use of these fungi as inoculants on agricultural crops.

## Materials and methods

### Plant material, fungus strain and inoculum preparation

Seeds of *L. multiflorum* Lam. and ECM fungus *X. badius* (Preservation No. cfcc5946) were obtained from the Xinrui Seed Industry Limited Company and China Forestry Culture Collection Center, respectively. Fungus maintenance, incubation, inoculation, and seeds pretreatment followed the methods of Liu et al.. (Liu et al. 2019).

### Effect of pH, salt, and drought stress on mycelial growth of *X. badius*

Three single-factor (pH, salt, or drought) experiments were performed. Five pH values, namely, 5.00, 6.00, 7.00, 8.00, and 9.00, were implemented to study the effect of pH on the mycelial growth of *X. badius.* Prior to sterilization, the pH level of the potato dextrose agar (PDA) medium was adjusted with an electronic pH meter (PHS-3C, INESA Ltd, Shanghai, China) by adding HCl (1.00 mol L^− 1^) or KOH (1.00 mol L^− 1^). Salt stress was imposed by adding 0.20% (w/v), 0.40% (w/v), 0.60% (w/v), and 0.80% (w/v) NaCl (corresponding to 34.22, 68.45, 102.67 and 136.89 mmol L^− 1^) to the PDA medium (pH = 6.50) before sterilization. *X. badius* growing at the absence of NaCl was used as the control. Drought stress was induced using 0.00% (w/v), 5.00% (w/v), 10.00% (w/v), 15.00% (w/v), and 20.00% (w/v) polyethylene glycol with a molecular weight of 6000 (PEG-6000) to adjust the water potential of the PDA medium (pH = 6.50) to approximately − 0.16, − 0.27, − 0.45, − 0.72, and − 1.07 MPa, respectively. As PEG reduces agar solidification, fungal isolates were grown in liquid medium (potato dextrose medium). To avoid submersion, a sterilized grit support was placed in the Petri dish with the liquid medium just covering the grit, and a fiber filter was placed on the grit with an inoculation on the filter.

All colonies were cultured in Petri dishes (diameter: 9.00 cm) filled with 10.00 mL of the modified culture medium as described above. Mycelial plugs with diameter of 5.00 mm were taken from the 7-day-old colony edge by using a sterilized mechanical puncher and transferred to the different tested media. At least six replicates were performed for each treatment. The inoculated Petri dishes were sealed with a strip of parafilm and maintained in the dark at 25.00 ± 1.00 °C and 60.00% relative humidity for 10 days in an incubator with constant humidity.

### Effect of ***X. badius*** inoculation on seed germination and seedling growth of ***L. multiflorum*** Lam. under salt and drought conditions

For each treatment, 30 *X. badius*-inoculated or non-inoculated seeds of *L. multiflorum* Lam. were sown in each Petri dish (diameter: 9.00 cm) with two layers of humid filter paper covered at the bottom. Two days after sowing, salt and drought were applied to the *X. badius*-inoculated and non-inoculated seeds. Salt stress was applied by adding 0.00% (w/v), 0.40% (w/v), and 0.80% (w/v) NaCl (according to the preliminary experiment) in the sterilized deionized water. Drought was imposed by adding 0.00% (w/v), 10.00% (w/v), and 20.00% (w/v) PEG-6000 in the sterilized deionized water. All Petri dishes were placed in a random position on a shelf in the laboratory. The experiment lasted for 2 weeks, during which all seedlings were watered every other day with NaCl, PEG-6000 solution, or sterilized water (control) and supplied twice a week with sterilized half-strength Hoagland’s solution (pH = 6.50) (Hoagland and Arnon 1950). In the meantime, the residual solution was poured out, and the filter papers were changed to avoid the effects of ion accumulation. To avoid edge effects, all Petri dishes were rotated weekly.

### Measurements of colony diameter (CD) and colony average growth rate (CGR)

After 7 days of incubation, the CD in different media was measured in the perpendicular direction using a beveled straightedge. The average of two diameter measurements along the perpendicular axes was used to estimate the colony size during the incubation period. The CGR was determined as the average increase in diameter divided by the total number of incubation days.

### **Measurements of seed germination rate (GR), shoot height (SH), and seedling total fresh weight (FW) **

One week after sowing, the cumulative number of germinated seeds in the different treatments was recorded, and the GR, which was defined as one hundred times the number of germinated seeds divided by the total number of seeds, was calculated. At the end of the experiment, the seedlings in the different treatments were harvested separately, washed in running tap water to remove the chemical substances, and divided into shoot and root portions. The SH and FW were measured.

### Statistical analyses

The experiments were performed using a completely randomized design. All the measurements were conducted in sextuplicate at least. Data were presented as mean ± standard deviation. Statistical analysis was carried out using the SPSS-13.0 for Windows (Standard released version 13.0 for Windows; SPSS Inc., IL, USA). One-way analysis of variance (ANOVA) was used to evaluate the effects of different pH values, salt concentrations and drought on mycelial growth of *X. badius*. Two-way ANOVA was used to evaluate the effects of *X. badius* inoculation and salt or drought stress on seed germination and seedling growth of *L. multiflorum* Lam.. Tukey’s honestly significant difference (HSD) post hoc test (*P* ≤ 0.05) was performed to test the existence of statistical differences for the same parameter among different treatments.

## Results

### Effect of pH on mycelial growth

One-way ANOVA showed that the pH level of the medium had no significant influence on the mycelial growth of *X. badius* (*P* > 0. 05, Table 1). *X. badius* mycelium had the ability to grow well at a wide pH range of 5.00 ~ 9.00. After 7 days of incubation, *X. badius* cultured in the medium with pH 8.00 showed the largest CD (7.14 cm) and the highest CGR (1.43 cm day^− 1^), and the smallest CD (6.83 cm) and lowest CGR (1.37 cm day^− 1^) were observed in medium with pH 5.00. However, statistical analysis showed no significant difference (*P* > 0.05) in the CD and CGR among the media with different pH levels.

**Table 1:** Influence of the medium pH on mycelial growth of *X. badius*

pH	Colony diameter (cm)	Colony average growth rate (cm day^− 1^)	One-way ANOVA
5.00	6.83 ± 0.25a	1.37 ± 0.05a	0.988
6.00	6.91 ± 0.35a	1.38 ± 0.07a	
7.00	7.00 ± 0.29a	1.40 ± 0.06a	
8.00	7.14 ± 0.31a	1.43 ± 0.06a	
9.00	6.89 ± 0.32a	1.38 ± 0.06a	

Data are presented as mean of at least six replicates ± standard deviation. Small letters in the same column show statistically significant differences among different pH treatments for the same parameter at *P* ≤ 0.05 based on Tukey’s HSD post hoc test

### Effect of salt stress on mycelial growth

The NaCl concentration of the culture medium had significant negative effect on the mycelial growth of *X. badius* (*P* < 0.001, Table 2). Significant differences in the CD and CGR of *X. badius* were observed among the media with different NaCl concentrations (*P* ≤ 0.05, Table 2). *X. badius* in the control medium (without NaCl) grew best, as manifested by the largest CD (7.56 cm) and highest CGR (1.51 cm day^− 1^). By contrast, the mycelial growth of *X. badius* in the presence of NaCl was significantly inhibited and decreased with increasing NaCl concentration. *X. badius* in 0.80% NaCl medium showed the smallest CD (5.83 cm) and lowest CGR (1.17 cm day^− 1^).

**Table 2:** Influence of NaCl-induced salt stress on mycelial growth of *X. badiu*s

NaCl concentration (%)	Colony diameter (cm)	Colony average growth rate (cm day^− 1^)	One-way ANOVA
0.00	7.56 ± 0.21a	1.51 ± 0.04a	45.669***
0.20	7.16 ± 0.26b	1.43 ± 0.05b	
0.40	6.68 ± 0.37c	1.34 ± 0.07c	
0.60	6.53 ± 0.28c	1.31 ± 0.06c	
0.80	5.83 ± 0.17d	1.17 ± 0.03d	

Data are presented as mean of at least six replicates ± standard deviation. Small letters in the same column show statistically significant differences among different NaCl-induced salt stress treatments for the same parameter at *P* ≤ 0.05 based on Tukey’s HSD post hoc test. ***Significant at *P* ≤ 0.001

### Effect of drought stress on mycelial growth

PEG-induced drought stress had significant effect on the mycelial growth of *X. badius* (*P* < 0.001, Table 3). The CD and CGR of *X. badius* in the control (− 0.16 MPa) were 8.27 cm and 1.18 cm day^− 1^, respectively. The CD (8.18 cm) and CGR (1.17 cm day^− 1^) of *X. badius* in 5.00% PEG (− 0.27 MPa) medium were not significantly different (*P* > 0.05) from those of the control. By contrast, the mycelial growth of *X. badius* was significantly inhibited by 10.00∼20.00% PEG-induced drought stress (− 0.45 ∼ − 1.07 MPa). Incubation in 20.00% PEG (− 1.07 MPa) medium produced the smallest CD (2.28 cm) and lowest CGR (0.33 cm day^− 1^).

**Table 3:** Influence of PEG-6000-induced drought stress on mycelial growth of *X. badius*

PEG-6000 concentration (%)	Colony diameter (cm)	Colony average growth rate (cm day^− 1^)	One-way ANOVA
0.00	8.27 ± 0.16a	1.18 ± 0.04a	96.365***
5.00	8.18 ± 0.09a	1.17 ± 0.05a	
10.00	6. 83 ± 0.12b	0.98 ± 0.07b	
15.00	5.89 ± 0.23c	0.84 ± 0.06c	
20.00	2.28 ± 0.43d	0.33 ± 0.03d	

Data are presented as mean of at least six replicates ± standard deviation. Small letters in the same column show statistically significant differences among different PEG-induced drought stress treatments for the same parameter at *P* ≤ 0.05 based on Tukey’s HSD post hoc test. ***Significant at *P* ≤ 0.001

### Effect of ***X. badius*** inoculation on seed germination of ***L. multiflorum*** Lam. under salt and drought conditions

*X. badius* inoculation (*P* ≤ 0.001), salinity (*P* ≤ 0.001) and their interaction (*P* ≤ 0.05) had significant effects on the GR (Table 4). In comparison with the non-saline treatment, the GRs of both non-inoculated and *X. badius*-inoculated *L. multiflorum* Lam. seeds were decreased by the NaCl-induced salt stress, and the non-inoculated seeds showed larger decrease in GR than the *X. badius*-inoculated ones. Compared with the non-saline condition, 0.40% and 0.80% NaCl induced 17.99% and 43.47% decrease in the GR of the non-inoculated seeds, respectively. The GRs of the *X. badius*-inoculated seeds decreased by 5.49 and 28.84% under 0.40% and 0.80% NaCl condition, respectively. Under non-saline condition, *X. badius* had no significant influence on the GR of *L. multiflorum* Lam., but the GR was enhanced by *X. badius* under 0.40% and 0.80% NaCl-induced saline conditions. Compared with the non-inoculated seeds, the GRs of the *X. badius*-inoculated seeds increased by 12.00% and 22.34% under 0.40% and 0.80% NaCl condition, respectively.

**Table 4:** Effect of *X. badius* inoculation on seed germination and seedling growth of *L. multiflorum* Lam. under different NaCl-induced salt conditions

	NaCl concentration (%)	Germination rate (%)	Shoot height (cm)	Seedling fresh weight (g)
Non-inoculated	0.00	93.29 ± 2.01a	22.61 ± 1.01a	5.64 ± 0.55b
	0.40	76.51 ± 1.74b	18.33 ± 1.54bc	4.76 ± 0.62c
	0.80	52.74 ± 4.63d	14.67 ± 1.63d	3.22 ± 0.43d
*X. badius*-inoculated	0.00	90.67 ± 1.89a	26.14 ± 1.29a	7.20 ± 0.18a
	0.40	85.69 ± 3.22ab	23.29 ± 1.22a	6.05 ± 0.39b
	0.80	64.52 ± 2.98c	18.83 ± 1.98bc	4.27 ± 0.47c
Two-way ANOVA				
Salt		34.710***	6.408**	383.828***
*X. badius*		18.866***	142.712***	372.925***
Salt × *X. badius*		3.272*	9.544***	10.846***

Data are presented as mean of six replicates ± standard deviation. Small letters in the same column show statistically significant differences among different salt stress treatments for the same parameter at *P* ≤ 0.05 based on Tukey’s HSD post hoc test. *, ** and ***Significant at *P* ≤ 0.05, 0.01, and 0.001, respectively

Compared with the non-drought condition, the PEG-induced drought decreased the GRs of both non-inoculated and *X. badius*-inoculated seeds, and the non-inoculated seeds showed larger decrease in GR than the *X. badius*-inoculated ones (Table 5). Compared with the non-drought condition, 10.00% PEG-induced drought stress led to 24.31% and 9.23% decrease in the GRs of the non-inoculated and *X. badius*-inoculated seeds, respectively. Meanwhile, 20.00% PEG-induced drought stress led to 49.32% and 37.20% decrease in the GRs of the non-inoculated and *X. badius*-inoculated seeds, respectively. Under non-drought condition, *X. badius* had no significant influence on the GR of *L. multiflorum* Lam., but *X. badius* enhanced GR of *L. multiflorum* Lam. under 10.00% and 20.00% PEG-induced drought conditions. Compared with the non-inoculated seeds, the GRs of the *X. badius*-inoculated seeds increased by 19.51% and 23.48% under 10.00% and 20.00% PEG condition, respectively.

**Table 5:** Effect of *X. badius* inoculation on seed germination and seedling growth of *L. multiflorum* Lam. under different PEG-6000-induced drought conditions

	PEG-6000 concentration (%)	Germination rate (%)	Shoot height (cm)	Seedling fresh weight (g)
Non-inoculated	0.00	92.18 ± 1.88a	23.22 ± 1.72b	5.58 ± 0.38b
	10.00	69.77 ± 1.89bc	16.45 ± 1.36cd	3.92 ± 0.39c
	20.00	46.72 ± 3.18d	13.86 ± 1.29d	2.74 ± 0.51d
*X. badius*-inoculated	0.00	91.86 ± 3.21a	27.76 ± 2.01a	7.19 ± 0.25a
	10.00	83.38 ± 2.77ab	21.39 ± 1.17b	5.36 ± 0.44b
	20.00	57.69 ± 3.26c	17.90 ± 1.43c	3.88 ± 0.71c
Two-way ANOVA				
Drought		41.099***	53.134***	121.025***
*X. badius*		12.247***	76.028***	115.298***
Drought × *X. badius*		7.418**	13.532***	4.952*

Data are presented as mean of six replicates ± standard deviation. Small letters in the same column show statistically significant differences among different drought stress treatments for the same parameter at *P* ≤ 0.05 based on Tukey’s HSD post hoc test. *, ** and ***Significant at *P* ≤ 0.05, 0.01, and 0.001, respectively

### Effect of ***X. badius*** inoculation on seedling growth of ***L. multiflorum*** Lam. under salt and drought conditions

**Fig. 1:**
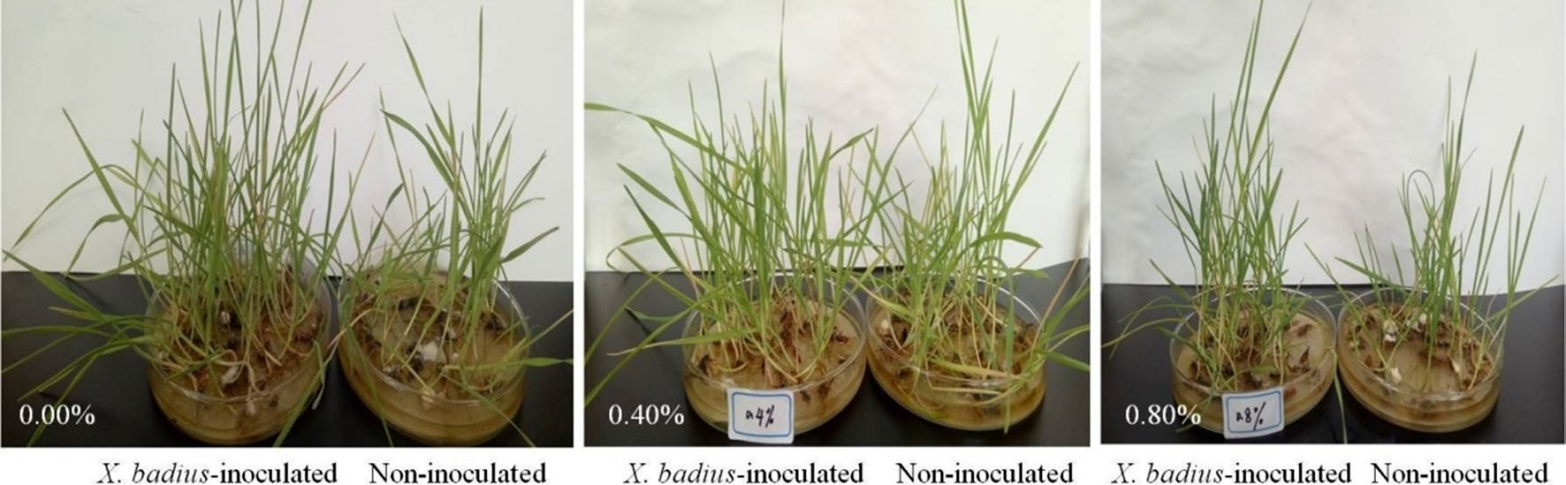
Typical phenotype of *L. multiflorum* Lam. seedlings 2 weeks after inoculation or non-inoculation with *X. badius* under different NaCl-induced salt conditions

*X. badius* inoculation, salinity, and their interaction had significant effects on the SH and FW (Table 4). Compared with those under the non-saline condition, salt stress inhibited the growth and biomass accumulation of non-inoculated and *X. badius*-inoculated *L. multiflorum* Lam. seedlings, and the non-inoculated seedlings showed a larger decrease than the *X. badius*-inoculated ones (Fig. 1). Compared with those under the non-saline condition, under 0.40% NaCl condition, the SHs of the non-inoculated and *X. badius*-inoculated seedlings decreased by 18.93% and 15.60%, respectively, while the FWs decreased by 10.90% and 15.97%, respectively. Under 0.80% NaCl condition, the SHs of the non-inoculated and *X. badius*-inoculated seedlings decreased by 35.12% and 42.91% respectively, while the FWs decreased by 27.96% and 40.69%, respectively. *X. badius* inoculation improved the SH and FW of *L. multiflorum* Lam. seedlings under non-saline and saline conditions. Under non-saline condition, *X. badius* increased the SH and FW by 15.61% and 27.66%, respectively. Under 0.40% and 0.80% NaCl condition, *X. badius* increased SH by 27.06% and 28.36%, respectively, while the FWs of the *X. badius*-inoculated seedlings increased by 27.10% and 32.61%, respectively.

**Fig. 2:**
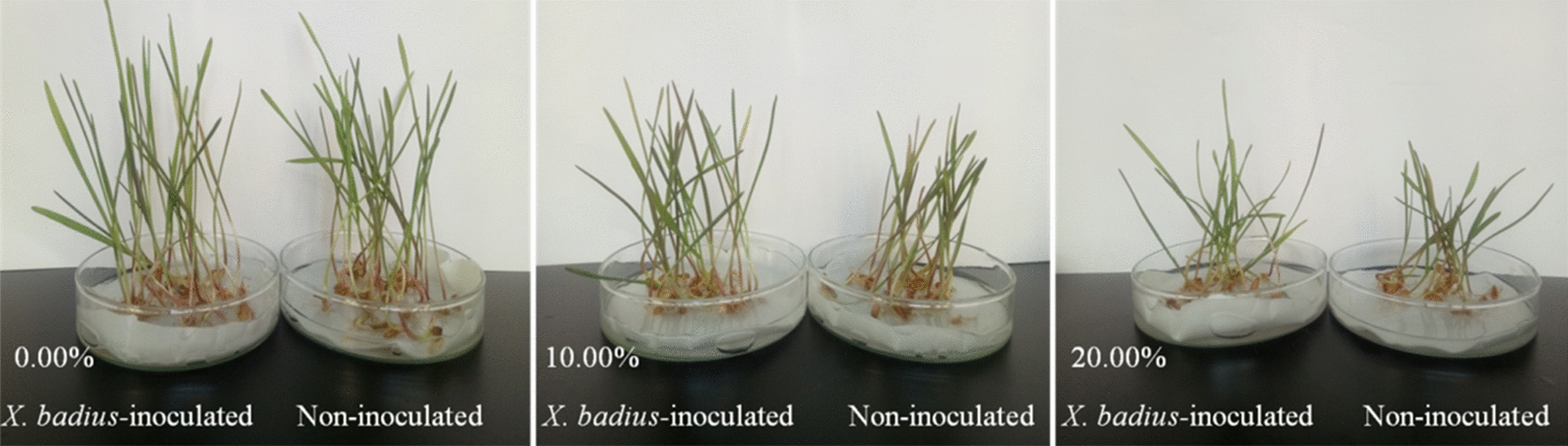
Typical phenotype of *L. multiflorum* Lam. seedlings 2 weeks after inoculation or non-inoculation with *X. badius* under different PEG-6000-induced drought conditions

*X. badius* inoculation, drought, and their interaction had significant effects on the SH and FW (Table 5). Compared with those under the non-drought condition, drought stress inhibited the growth and biomass accumulation of non-inoculated and *X. badius*-inoculated *L. multiflorum* Lam. seedlings, and the non-inoculated seedlings showed a larger decrease than the *X. badius*-inoculated ones (Fig. 2). Compared with those under the non-drought condition, the SHs of the non-inoculated and *X. badius*-inoculated seedlings decreased by 29.16% and 22.95%, respectively, under 10.00% PEG condition and by 40.31% and 37.36%, respectively, under 20.00% PEG condition. The FWs of the non-inoculated and *X. badius*-inoculated seedlings decreased by 29.75% and 25.45%, respectively, under10.00% PEG condition and by 50.90% and 46.04%, respectively, under 20.00% PEG condition. *X. badius* inoculation improved the SHs and FWs of the *L. multiflorum* Lam. seedlings under non-drought and drought stress conditions. Compared with those of the non-inoculated seedlings, the SHs of the *X. badius*-inoculated seedlings increased by 19.55%, 30.03%, and 29.15% under 0.00%, 10.0%, and 20.00% PEG-induced drought condition, respectively, and the FWs increased by 28.86%, 36.73%, and 41.61% under 0.00%, 10.00%, and 20.00% PEG-induced drought condition, respectively.

## Discussion

### Effect of pH on mycelial growth

The pH level is one of the crucial factors affecting the mycorrhizal fungus growth and development mainly by influencing the nutrient availability of the culture medium (Daza et al. 2006; Lazarević et al. 2016; Xu et al. 2008). ECM fungi can grow under conditions from acidic to slight alkaline (Zhu et al. 2008; Siri-in et al. 2014), but each fungal species has its optimum pH level for mycelial growth (Lazarević et al. 2016). For example, the mycelium of *Scleroderma sinnamariense* can grow at a pH range of 2.00 ~ 9.00, with the optimal pH of 5.00 (Siri-in et al. 2014). *Boletus edulis* and *Hebeloma* sp. showed the largest CD at pH 5.00, and *Laccaria bicolor* and *Laccaria deliciosus* grew best at pH 6.00 (Xu et al. 2008). The optimum pH levels of the aforementioned fungi were lower than 6.00, suggesting a good adaption to acid conditions. However, fungal species, such as *Amanita caesarea* (Daza et al. 2006), *Laccaria insulsus* (Xu et al. 2008), and some pleosporalean fungi from saline areas (Qin et al. 2017), grow best at neutral or slightly alkaline conditions. *X. badius* was isolated from soils with a pH range of 6.50 ~ 7.50. The colony may grow well in a culture medium with a pH level similar to its natural soil environments. Therefore, the pH conditions of the soil from which the fungi are isolated should be considered to optimize the culture and propagation of the fungi in the laboratory and to improve the production of mycorrhizal plants in the nursery. The results indicated that the mycelium of *X. badius* could grow well at the wide pH range of 5.00 ~ 9.00 (Table 1). After 7 days of incubation, *X. badius* grown at pH 8.00 showed the largest CD and the highest CGR, and the smallest CD and the lowest CGR were observed at pH 5.00. However, no significant difference (*P* > 0.05) were found in the CDs and CGRs among the media with different pH values (Table 1). *X. badius* might present high resistance under alkaline conditions, and this characteristic is typical of alkalophilic fungal species (Kulkarni et al. 2019).

### Effect of salt stress on mycelial growth

Salt stress is one of the most important limiting factors in agriculture worldwide. The practical use of beneficial mycorrhizal fungi with high salt tolerance has been proved to be one of the most effective strategies to alleviate the adverse effects on crops in saline areas (Guerrero-Galán et al. 2019; Kumar and Verma 2018). Salt-tolerance evaluation of mycorrhizal fungi in the laboratory could provide a useful theoretical reference for the selection of the proper fungal strain. In this study, *X. badius* was very sensitive to salt stress, although the mycelium also grew very well, that is consistent with observations on other fungi (Qin et al. 2017; Tang et al. 2009). Mycelial growth, as reflected by CD and CGR, was significantly inhibited with increasing NaCl concentration (*P* < 0.001, Table 2). *X. badius* in the non-saline medium grew best as manifested by the highest value in CD and CGR. *X. badius* in 0.80% NaCl medium showed the lowest value in CD and CGR, suggesting the worst growth performance (Table 2). Probably, *X. badius* had poor ability to absorb Na^+^ and Cl^−^, and the accumulation of these redundant ions in the medium resulted in low water potential and then reduced the availability of nutrient and water for the fungi (Kumar and Verma 2018), thereby leading to the restriction of mycelial growth. Despite its salt sensitivity, *X. badius* could still grow and survive in 0.80% NaCl medium, suggesting that this species is more likely halotolerant but not halophilic.

However, in nature, soil salinity is caused not only by NaCl but also by magnesium, calcium, potassium, etc. (Chen et al. 2019). More future researches focused on the effect of natural soil salinity on the growth of mycelia and the host plant should be carried out, that have more realistic significance in the utilization of salinity soil.

### Effect of drought stress on mycelial growth

Researches on the effect of PEG-induced drought stress on mycelial growth have been carried out with many ECM fungal strains (Navarro-Ródenas et al. 2011; Zhang et al. 2011; Zhu et al. 2008). In this study, the growth response of *X. badius* to drought stress induced by PEG-6000 was assessed. The results showed that 5.00% PEG-induced drought stress had no significant negative influence on the CD and CGR of *X. badius* (*P* > 0.05, Table 3). However, the mycelial growth of *X. badius* was significantly inhibited under 10.00% ~ 20.00% PEG-induced drought conditions as manifested by the significant decrease in CD and CGR (*P* ≤ 0.05, Table 3). Mycelial growth under water-controlled conditions could reflect the adaptability of fungus to dry soil and the ability of the fungus to enhance the drought resistance of its host plants (Duñabeitia et al. 2004). Also, host plants may influence the morphology and physiology of the fungus after mycorrhization (Zhang et al. 2011). Therefore, it is necessary to establish fungus-mycorrhiza-host plant symbiont and study the associating drought resistance prior to practical application.

### Effect of seed priming with ***X. badius*** suspensions on seed germination and seedling growth of ***L. multiflorum*** Lam. under salt and drought conditions

Drought and high salinity are the two most important environmental factors that adversely affect the seed germination of crops and the survival, growth, and productivity of plants. In recent years, seed bio-priming with PGPF spore suspensions has been extensively proved to be beneficial for the seed germination and seedling growth of crops under non-stress and stress conditions (Bonfante and Genre 2010; de Zelicourt et al. 2013; Guerrero-Galán et al. 2019; Hossain et al. 2017; Javeria et al. 2017; Kumar and Verma 2018; Tomer et al. 2016; Vijayabharathi et al. 2016; Vimal et al. 2017; Yan et al. 2019). Based on the findings from the tolerance test of *X. badius* and the verified mutualistic symbiosis between *L. multiflorum* Lam. and *X. badius* driven by seed inoculation (Liu et al. 2019), the effect of seed priming with spore suspensions of *X. badius* on seed germination and seedling growth of *L. multiflorum* Lam. were investigated under different salt and drought conditions. The results indicated that seed priming with *X. badius* had no significant effect on the GR under non-stress condition (*P* > 0.05, Tables 4 and 5), that is consistent with our previous study (Liu et al. 2019) and studies on bromeliad (Leroy et al. 2019), barley and oat (Murphy et al. 2017) inoculated by other PGPF species. However, GR was significantly enhanced by seed priming with *X. badius* under drought and salt stress conditions. *X. badius* inoculation greatly improved the SH and FW of *L. multiflorum* Lam. seedlings under non-stress and drought/salt stress conditions (Figs. 1 and 2). The improvement under stress conditions was markedly higher than that under non-stress conditions (*P* ≤ 0.05, Tables 4 and 5). Similar improvements in seed germination and seedling growth induced by mycorrhizal fungi inoculation with seeds have also been reported on *Dendrobium officinale* (Tan et al. 2014) and other epiphytic orchid species (Alghamdi 2019). The results also showed that *X. badius* inoculation led to earlier seed germination and greater survival of seedlings compared with the non-inoculated seeds under non-stress and stress conditions. Thus, fungal inoculation with seeds was not very essential for seed germination under non-stress and mild stress conditions but contributed highly to the survival and growth of the seedlings especially under severe stress conditions. The symbiotically associated fungi could promote the degradation of the cuticle cellulose of the seed resulting in the alleviated restriction of seed coat and then earlier germination. In addition, it can also produce many plant growth-promoting compounds such as phytohormones (gibberellins and indole acetic acid) and secondary metabolites, and enhance water and nutrient availability, which are conducive to seed germination and subsequent seedling growth (Behie and Bidochka 2014; Cairney 2012; Garcia et al. 2016; Hossain et al. 2017; Javeria et al. 2017; Owen et al. 2015; Shen et al. 2018).

In comparison with the non-stress condition, NaCl-induced salt stress and PEG-induced drought stress decreased GR, SH, and FW of the non-inoculated and *X. badius*-inoculated seeds/seedlings, and the non-inoculated seeds/seedlings showed larger decrease in these three parameters than the *X. badius*-inoculated ones (Figs. 1 and 2; Tables 4 and 5). The GRs, SHs, and FWs of both non-inoculated and *X. badius*-inoculated *L. multiflorum* Lam. seeds/seedlings decreased rapidly with the increase of NaCl and PEG concentrations, and PEG showed more negative effect than that of NaCl (Tables 4 and 5), which is in agreement with the results from previous studies (Murillo-Amador et al. 2002; Petrović et al. 2016). The inhibition by salt and drought stress on seed germination was mainly due to the limited water uptake by the seed, which caused the subsequent inhibition on the seedling growth. Probably, the accumulation of Na^+^ and Cl^−^ in the substrate could also result in the toxic effect on seed germination and seedling growth by creating an external osmotic potential (Zhang et al. 2010). Compared with that under the PEG solution, the osmotic potential difference caused by the ion accumulation in the NaCl solution can also induce the rapid water uptake of seed and thereby enough water content for earlier seed germination.

In conclusion, the experimental evidence of the ability of *X. badius* to adapt to a series of environmental stresses, including pH, salt stress, and drought stress, is presented. The results indicated that *X. badius* had a wide pH tolerance, especially high alkali tolerance, and might has good adaptation to alkali environments. Furthermore, seed priming with spore suspensions of *X. badius* was not essential to the seed germination of *L. multiflorum* Lam. under non-stress and mild stress conditions, but induced a beneficial effect on the subsequent seedling growth under severe salt and drought stress conditions. Hence, the successful establishment of *X. badius* on *L. multiflorum* Lam. seedlings under stressful conditions can be an effective approach to increase the plant tolerance to withstand environmental stresses.

## Data Availability

The authors declare that all the data and materials used in this study are available.
